# Round-robin test on thermal conductivity measurement of ZnO nanofluids and comparison of experimental results with theoretical bounds

**DOI:** 10.1186/1556-276X-6-258

**Published:** 2011-03-25

**Authors:** Wook-Hyun Lee, Chang-Kyu Rhee, Junemo Koo, Jaekeun Lee, Seok Pil Jang, Stephen US Choi, Ki-Woong Lee, Hwa-Young Bae, Gyoung-Ja Lee, Chang-Kyu Kim, Sung Wook Hong, Younghwan Kwon, Doohyun Kim, Soo Hyung Kim, Kyo Sik Hwang, Hyun Jin Kim, Hyo Jun Ha, Seung-Hyun Lee, Chul Jin Choi, Ji-Hwan Lee

**Affiliations:** 1 Energy Efficiency and Materials Convergence Research Division, Korea Institute of Energy Research, Daejeon, Republic of Korea; 2 Nuclear Materials Research Division, Korea Atomic Energy Research Institute, Daejeon, Republic of Korea; 3 Department of Mechanical Engineering, Kyung Hee University, Yongin, Republic of Korea; 4 Department of Mechanical Engineering, Pusan National University, Busan, Republic of Korea; 5 School of Aerospace and Mechanical Engineering, Korea Aerospace University, Republic of Korea; 6 Mechanical and Industrial Engineering Department, University of Illinois at Chicago, Chicago, IL, USA

## Abstract

Ethylene glycol (EG)-based zinc oxide (ZnO) nanofluids containing no surfactant have been manufactured by one-step pulsed wire evaporation (PWE) method. Round-robin tests on thermal conductivity measurements of three samples of EG-based ZnO nanofluids have been conducted by five participating labs, four using accurate measurement apparatuses developed in house and one using a commercial device. The results have been compared with several theoretical bounds on the effective thermal conductivity of heterogeneous systems. This study convincingly demonstrates that the large enhancements in the thermal conductivities of EG-based ZnO nanofluids tested are beyond the lower and upper bounds calculated using the models of the Maxwell and Nan et al. with and without the interfacial thermal resistance.

## Introduction

Nanofluids, a new class of fluids engineered by uniformly dispersing nanostructures such as nanoparticles, nanotubes, nanorods, and nanofibers, in base fluids, have heat and mass transport properties that are far superior to those of the base fluids. For example, a number of research groups presented surprising experimental findings that nanofluids significantly enhance thermal conductivities [1-8], convective heat transfer coefficient [9-13], and heat absorption rate [14]. Therefore, these novel nanofluids have the potential to become next-generation coolants and working fluids for innovative applications in industries such as energy, bio and pharmaceutical industry, and chemical, electronic, environmental, material, medical and thermal engineering among others [15,16]. Nanofluids have thus attracted considerable interest worldwide. Hundreds of research groups, in both academia and industry, are exploring nanofluids. Most recently, the European Commission launched Nanohex [17], the world's largest collaborative project for the research and development of nanofluid coolants, bringing together 12 partners from academia and industry, ranging from small- and medium-sized enterprises (SMEs) to global companies such as Siemens and Thermacore.

Of all the properties of nanofluids, thermal conductivity has sparked the most excitement and controversy. The anomalous enhancement of measured thermal conductivity [1-8], as compared with the predictions of the classical models, has generated excitement in both academia and industry. However, these data became controversial years later when no anomalous enhancement in thermal conductivity was observed [18-20]. These contradictory data have generated another controversy regarding the mechanisms of enhanced thermal conductivity in nanofluids. For example, a number of investigators proposed that new mechanisms are needed to explain anomalous enhancement [21-26]. However, some others [27-29] show that the thermal conductance mechanism in nanofluids is no different from that in binary solid composites or liquid mixtures, and that thermal conductivity data lie between the well-known effective medium bounds of the Hashin and Shtrikman (H-S) [30]. But, Murshed [31] pointed out that more systematic and careful investigations are needed to resolve the controversy over the mechanism of the enhanced thermal properties. Moreover, Schmidt et al. [32] showed that the thermal conductivity of nanofluids is greater than the Hamilton-Crosser model [33].

These contradictory thermal conductivity data highlight the need for more controlled synthesis and accurate characterization of nanofluids. One way to reduce data inconsistencies due to differences in sample quality, such as particle size and size distribution including agglomeration, is to conduct round-robin tests using identical test samples. Recently, Buongiorno et al. [34] launched an International Nanofluid Property Benchmark Exercise (INPBE) to resolve the inconsistencies in the database. They reported that the nanofluids tested in INPBE exhibit thermal conductivity in good agreement with the predictions of the effective medium theory for well-dispersed nanoparticles.

There are several reasons for the good agreement. First, the nanofluids used in the INPBE were manufactured by two-step method with surfactant (Set 1) and chemical reduction method with several electrolytes (Set 2) or commercial products with various surfactants and electrolytes (Sets 3 and 4). Second, measurement uncertainty analysis is essential because the measured thermal conductivity data may have biases and random variation. However, most organizations using transient hot wire method (THWM) for measurement of the thermal conductivity did not perform the measurement uncertainty analysis.

So we thought that it would be interesting to produce nanofluids by a one-step physical method with no surfactant, perform measurement uncertainty analysis, and measure the thermal conductivity of the nanofluids using very accurate thermal conductivity apparatuses.

The objectives of this study are to conduct a round-robin test on thermal conductivity measurements of three samples of EG-based ZnO nanofluids and compare the experimental results with theoretical bounds on the effective thermal conductivity of heterogeneous systems.

Different methods of sample preparation or even small differences in the sample preparation process can cause large differences in sample properties. Therefore, in this study, one laboratory synthesized all three samples of ZnO nanofluids using one-step pulsed wire evaporation (PWE) process to be described in "Synthesis of ZnO nanofluids" section. The round-robin exercise involved five test-laboratories that have extensive experience in the thermal conductivity measurement of nanofluids. Each participant received identical samples of ZnO nanofluids and was asked to conduct the test within 2 weeks of receipt of samples. The five participating laboratories measured the thermal conductivity of the samples of ZnO nanofluids over a temperature range from 20 to 90°C using the THWM. The results were collected, analyzed, and plotted for comparison with several theoretical bounds [30,35,36] on the effective thermal conductivity of heterogeneous systems.

Based on the results of these round-robin tests using identical test samples synthesized by one-step PWE method and accurate thermal conductivity apparatus with measurement uncertainty <1.5%, we clearly show that the large enhancements in the thermal conductivity of the EG-based ZnO nanofluids are beyond the lower and upper bounds of both the Maxwell model [35] with and without the interfacial thermal resistance and the Nan et al. model [36].

## Experiments

### Synthesis of ZnO nanofluids

Various synthesis procedures have been used for production of nanofluids. The PWE method is one approach to fabricate nanoparticles [37]. In this study we used the PWE method mainly because the process is simple to use, and it is not time consuming to produce nanofluids samples in enough quantity for the round-robin measurements.

Although the thermal conductivity of suspensions of ZnO nanoparticles in water or EG was studied, the previous studies [38-44] used nanofluids manufactured by the two-step method or commercial products with surfactants, as shown in Table 1. However, in this study, the EG-based ZnO nanofluids are manufactured by a one-step physical method using PWE [37] and do not contain any surfactant. Therefore, the ZnO nanofluids studied in this work are different from the previously studied ZnO nanofluids [38-44].

**Table 1:** Previous studies on EG/water-based ZnO nanofluids

Paper	Manufacturing method	Measurement method (Accuracy)	Base fluid	Surfactant	Comments
Kim et al. [38]	Two-step	THW (1%)	Water/EG	Sodium dodecyl sulfate (SDS) of 0.05 M	Size dependence

Yu et al. [39]	Two-step	STHW (1%)	EG	-	No temperature dependence

Moosavi et al. [40]	Two-step	KD2 Pro (5%)	EG/glycerol	Ammonium citrate (dispersant:nanoparticle = 1:1 wt.%)	Temperature dependence

Raykar and Singh [41]	Two-step	THW	Water	3, 5, and 7 mL of acetylacetone (acac) is added in type I, II, and III solutions	Temperature dependence without low vol. %

Shen [42]	Commercial high-volume fraction dispersions in water with chemical dispersant (Nanophase)	THW	Water	Addition of chemical dispersants which is not disclosed by the company	Reverse size dependence

Vajjha and Das [43]	Commercial 50% dispersion in water (Alfa Aesar)	Commercial device based on steady-state method^a^ (2.45%)	EG:W (6:4 wt.%)	Dispersant not clear	Temperature and size dependence

Xie et al. [44]	Two-step	-	EG:W (45:55 vol.%)	-	

^a^Experimental Operating and Maintenance Procedures for Thermal Conductivity of Liquids and Gases Unit, P.A. Hilton Ltd., Hampshire, England. THW, transient hot wire, STHW, short transient hot wire, EG ethylene glycol.

**Figure 1:**
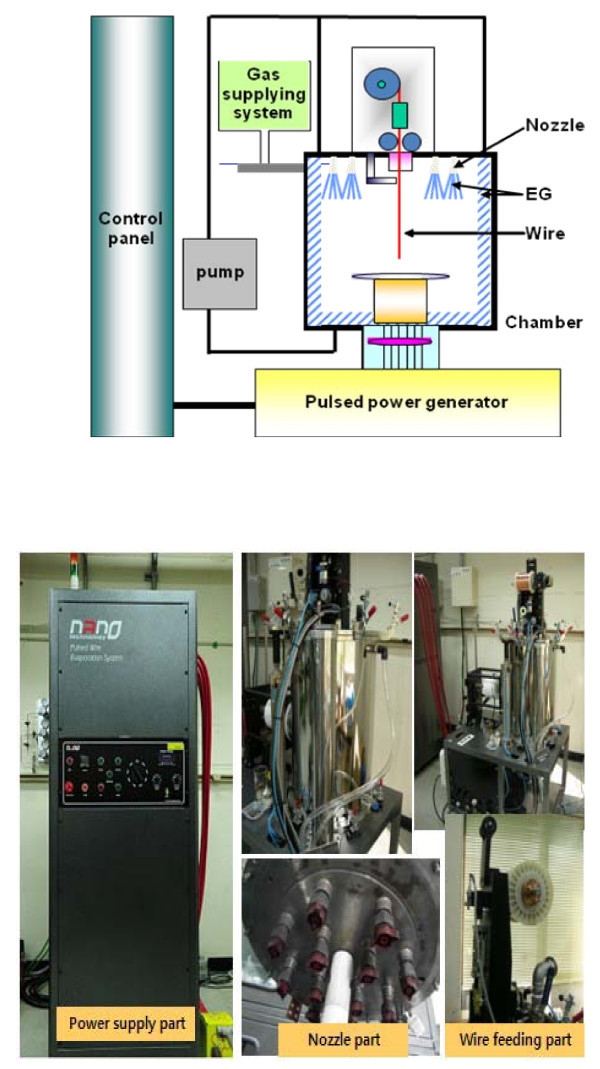
**Schematic diagram and photographs of PWE system and subsystems**. **(a) **Schematic diagram of PWE system. **(b) **Photograph of PWE subsystems.

As shown in Figure 1, the PWE system for synthesis of EG-based ZnO nanofluids consists of three main components which are the pulsed power generator, the control panel, and the evaporation chamber with continuous wire feeding and fluid nozzle subsystems. Pure Zn wire of 99.9% with a diameter of 0.5 mm was used as a starting material and the feeding length of the wire into the reaction chamber was 100 mm. When a pulsed high voltage of 25 kV is driven through a thin wire, non-equilibrium overheating induced in the wire makes the wire evaporate into plasma within several microseconds. Then the high-temperature plasma is cooled by an interaction with an argon-oxygen mixed gas, and evaporated Zn gas is condensed into small-sized particles and spontaneously immersed into EG-stained chamber. The Ar:O_2 _atmosphere in the evaporation chamber facilitates formation of the zinc oxide phase. More details of the PWE method and system are given in [45].

**Figure 2:**
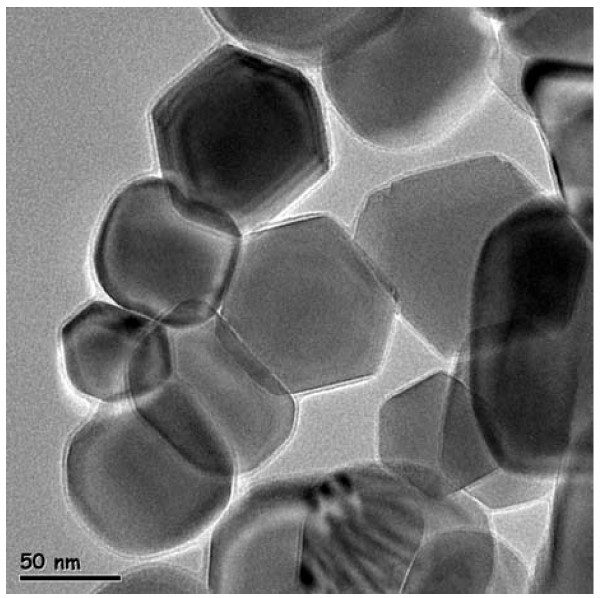
**TEM image of ZnO nanoparticles produced by PWE process**.

Using the one-step PWE process, three test samples were produced: EG-based ZnO nanofluids with nanoparticle concentrations of 1.0, 3.0, and 5.5 vol.%. A transmission electron microscopy (TEM) image of ZnO nanoparticles with an average diameter of 70 nm is shown in Figure 2.

### Thermal conductivity measurements and uncertainty analysis

In this study, four of the labs used a THWM developed in house to measure the thermal conductivity of EG-based ZnO nanofluids, and one of the five labs performed the thermal conductivity measurements using a commercial apparatus, LAMBDA (LAMBDA F5 Technology, Germany) with 1% error.

**Figure 3:**
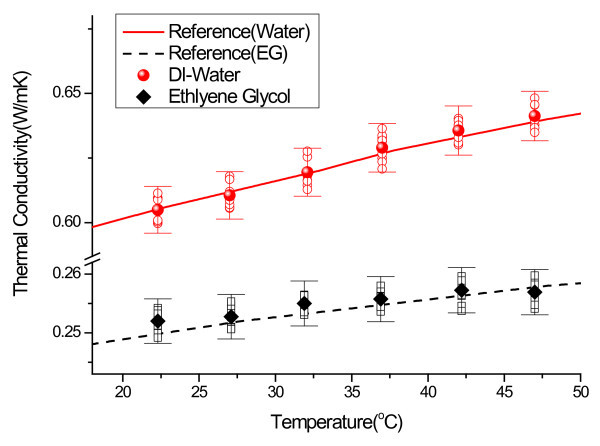
**Validation of transient hot wire apparatus**.

In order to obtain the accuracy of the transient hot wire apparatus, the measurement uncertainty analysis of the apparatus was performed by each laboratory as follows:

The thermal conductivity of fluids is calculated by Equation 1,(1)

where *k, q, ΔT *and *t *are the thermal conductivity, the input power per unit length, the temperature rise of hot wire, and the measurement time, respectively. The thermal conductivity of fluids can be obtained if the input power unit length and temperature rise of hot wire are measured as a function of temperature. Therefore, the measurement uncertainty of the apparatus [46] is given by Equation 2,(2)

where *u_k_, u_q_*, and *u*Δ_*T *_are the measurement uncertainties of thermal conductivity, the input power per unit length, and the temperature rise of the hot wire, respectively. Equation 2 shows that the measurement uncertainty of the thermal conductivity using the transient hot wire apparatus consists of the measurement uncertainties of input power per unit length, *q*, and the temperature rise of hot wire, Δ*T*. Here the measurement uncertainties of *q *and Δ*T *in accordance with 95% confidence interval [47,48] are expressed by Equation 3,(3)

where *u_i_, B*, and *t_λ,95%_P *are the measurement uncertainty of *i*, bias error, and estimate of the precision error in the repeated measurement data at 95% confidence. In addition, *λ *is the degree of freedom given by,(4)

where *N *is the data size. Using this method, the measurement uncertainty of transient hot wire apparatus manufactured by each lab was determined to be less than 1.5%. In order to verify the accuracy and the reliability of this experimental system, the thermal conductivity was experimentally measured using deionized water and EG. As shown in Figure 3, a typical THW apparatus calibration with the reference fluids demonstrates that it is possible to measure thermal conductivities with less than 1.5% error, verifying the estimated measurement uncertainty of 1.5%. In Figure 3, the hollow symbols represent the calibration data and the solid symbols are the average value of the calibration data. The solid and dashed lines represent the thermal conductivity of water and EG, respectively [49].

## Results and discussion

### Results of the round-robin study and statistical treatment of data

**Figure 4:**
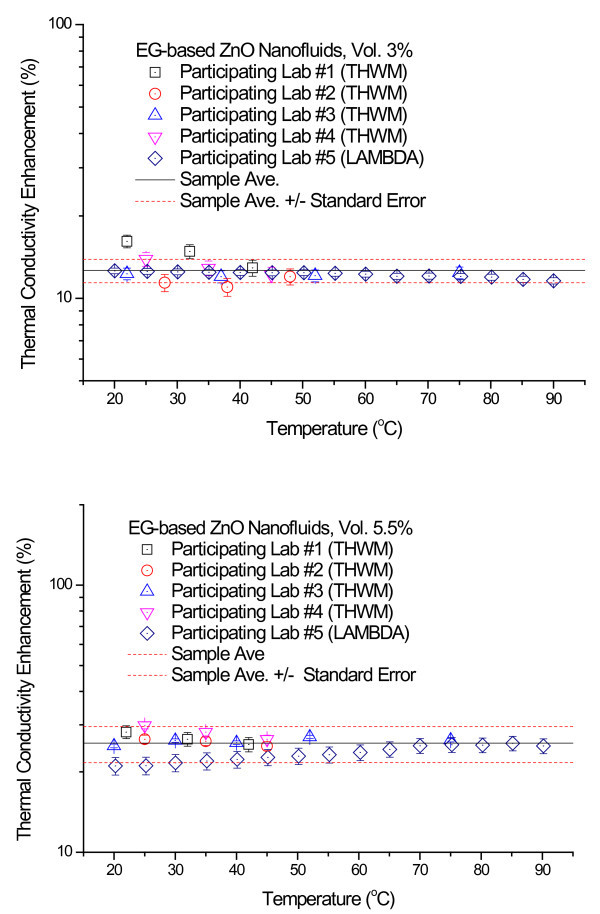
**Thermal conductivity enhancement for the 3.0 vol.% and 5.5 vol.% ZnO nanofluids as a function of temperature**. **(a) **Thermal conductivity enhancement data for 3.0 vol.% ZnO nanofluids. **(b) **Thermal conductivity enhancement data for 5.5 vol.% ZnO nanofluids.

Figure 4a, b shows the thermal conductivity enhancements for the 3.0 and 5.5 vol.% ZnO nanofluids that were measured at each of the five participating labs. The thermal conductivity enhancement is defined as (*k*_eff _- *k*_f_)/*k*_f_, where *k*_eff _and *k*_f _are the thermal conductivity of nanofluids and base fluids, respectively. Each data point represents the ratio of the mean of 10 measured enhancements to the thermal conductivity of base fluid. Error bars show measurement uncertainty determined by the participating labs as described in the previous section. Figure 4a, b indicates that the experimental thermal conductivity data show very little dependence on temperature in the 20 to 90°C range.

Following the statistical data analysis procedures used in the INPBE study [34], we calculated the sample averages and the standard errors for all the thermal conductivity enhancement data. In Figure 4a, b, the sample average is shown as a solid line and the standard errors of the sample mean as dotted lines. As seen in Figure 4a, b, the experimental data obtained by the five participating labs lie within a narrow band about the sample average with only a few modest outliers. The data analysis shows that the standard errors of the sample mean for the 3.0 and 5.5 vol.% ZnO nanofluids samples are ±1.24 and ±3.95%, respectively.

**Figure 5:**
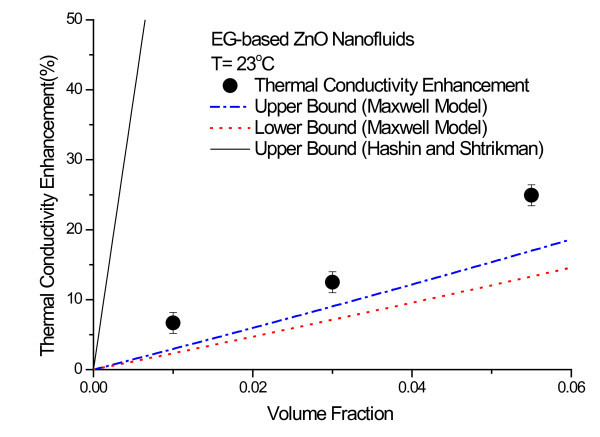
**Thermal conductivity enhancement of EG-based ZnO nanofluids as a function of nanoparticle volume fraction**.

Figure 5 shows the thermal conductivity enhancement of EG-based ZnO nanofluids at a temperature of 23°C as a function of nanoparticle volume fraction. Each data point represents the ratio of the ensemble average of enhancements measured by the participating labs at a given volume fraction to the thermal conductivity of base fluid. The error bars show the standard deviation from the ensemble average. The ZnO nanofluids show very significant increases in thermal conductivity, with a nearly 25% increase for 5.5 vol.% ZnO nanoparticles.

### Comparison of experimental results with theoretical bounds

The Hashin and Shtrikman (H-S) bounds on the thermal conductivity of heterogeneous systems [30] have been used for nanofluids to show that the effective medium theory can explain the enhancement of nanofluids [27,29]. The H-S upper bound is given by Equation 5 and the H-S lower bound is the classical Maxwell model as given by Equation 6. Recently, Buongiorno et al. [34] used Equation 6, the classical Maxwell model with negligible interface resistance, for the upper bound for nanofluids and Equation 7, the Maxwell model with interface resistance, for the lower bound for nanofluids.

Upper bound of Hashin and Shtrikman [30](5)

Upper bound of the Maxwell Model (no interfacial thermal resistance) [35](6)

Lower bound of the Maxwell Model (with interfacial thermal resistance) [50](7)

where *k*_f_, *k*_p_, *r*_p_, *R*_b_, and *φ *are the thermal conductivities of base fluids and nanoparticles, radius of nanoparticles, interfacial thermal resistance, and volume fraction of nanoparticles, respectively.

**Figure 6:**
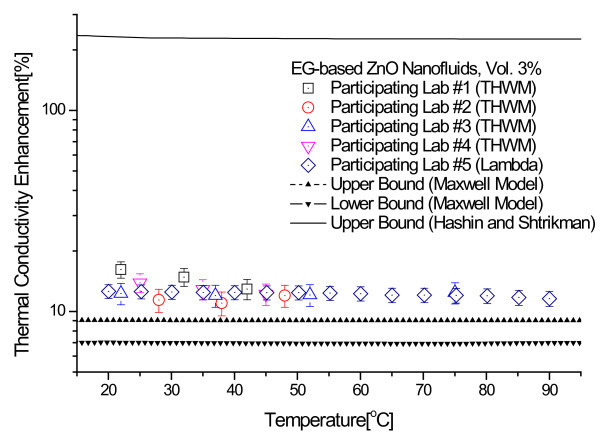
**Comparison of experimental thermal conductivity enhancements of 3.0 vol.% ZnO nanofluids with theoretical bounds of H-S and Maxwell models**.

**Figure 7:**
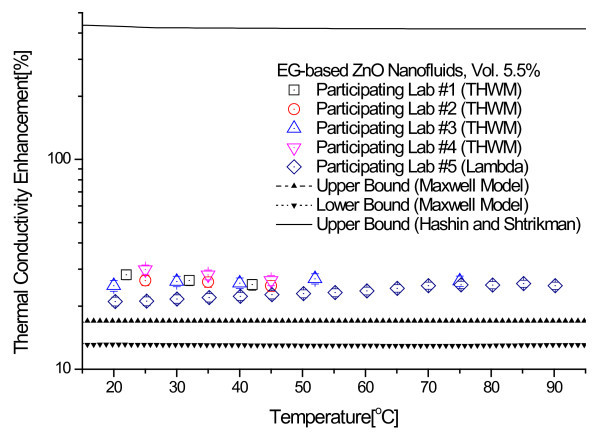
**Comparison of experimental thermal conductivity enhancement of 5.5 vol.% ZnO nanofluids with theoretical bounds of H-S and Maxwell models**.

Figures 6 and 7 show comparisons of experimental thermal conductivity enhancements of 3.0 vol.% and 5.5 vol.% ZnO nanofluids with the three theoretical bounds of Hashin and Shtrikman and Maxwell models. The properties, such as the thermal conductivities of EG [49] and ZnO nanoparticles [51], used for calculating the theoretical bounds are summarized in Table 2. The upper bound of Hashin and Shtrikman, which was used by Eapen et al. [27] and Kelbinski et al. [29], dramatically overestimates the thermal conductivity of ZnO nanofluids. The H-S upper bound corresponds to large pockets of fluid separated by linked chain-forming or clustered nanoparticles [29]. The long wire-like structures made of perfectly aligned nanoparticles are not realizable with dilute nanofluids with well-dispersed nanoparticles. Furthermore, it is almost impossible to separate fluid by nanoparticle chains in nanofluids, although nanoparticles can be partially aggregated in nanofluids. Therefore, the upper bound given by the H-S model is not applicable to nanofluids. More realistic upper and lower bounds for nanofluids having low concentration of well-dispersed nanoparticles are given by Buongiorno et al. [34]. It is clear from Figures 6 and 7 that the thermal conductivity enhancements of EG-based ZnO nanofluids are larger than the upper bound of the Maxwell model.

**Table 2:** Material properties used to calculate theoretical bounds

	*φ *(%)	*r*_p _(nm)	***k*_f _(W/mK) **[49]	***k*_p _(W/mK) **[51]	*R*_b _(m2K/W)
					**Upper bound/lower bound **[52]
Sample 1	1.0		280	0.244	
			290	0.248	
			300	0.252	
Sample 2	3.0	35	310	0.255	29 0/10^-8^
			320	0.258	
			330	0.260	
			340	0.261	
Sample 3	5.5		350	0.261	
			360	0.261	
			370	0.262	

In addition, we used the generalized Maxwell model developed by Nan et al. [36] with and without interfacial resistance for the lower and upper bounds for nanofluids. The Nan et al. model is given in Equation 8.

Nan et al. [36] model(8)

, , , , 

where *a*_ii_, *a*_k_, *L*_ii_, p, *φ*, and  are the diameter of the ellipsoid, Kapitza radius, geometrical factors dependent on the particle shape, aspect ratio of the ellipsoid, volume faction, and equivalent thermal conductivities, respectively. *R*_bd _is the interfacial thermal resistance, also known as thermal boundary resistance, or Kapitza resistance.

**Figure 8:**
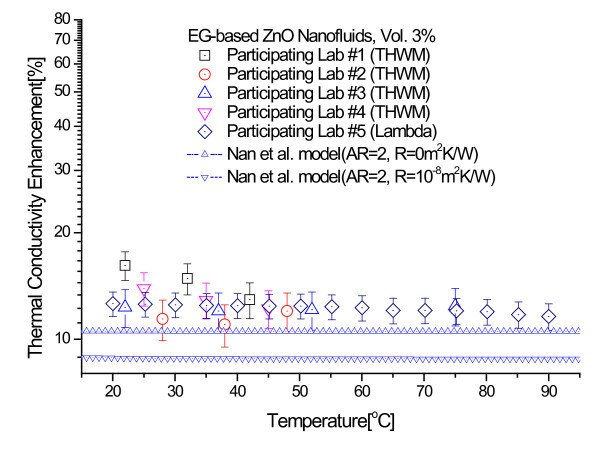
**Comparison of experimental thermal conductivity enhancements of 3.0 vol.% ZnO nanofluids with theoretical bounds of Nan et al. model**.

**Figure 9:**
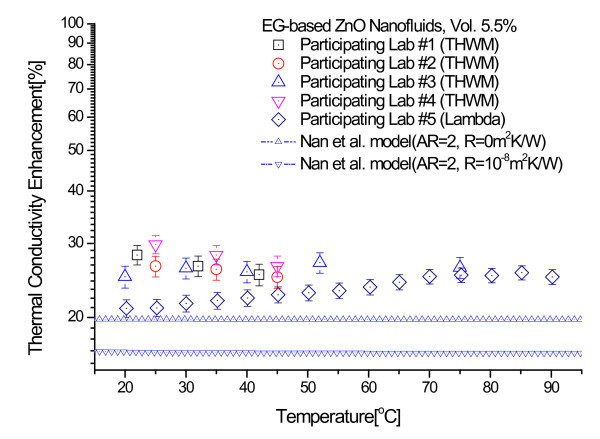
**Comparison of experimental thermal conductivity enhancements of 5.5 vol.% ZnO nanofluids with theoretical bounds of Nan et al. model**.

Figures 8 and 9 show comparisons of the experimental thermal conductivity enhancements of 3.0 and 5.5 vol.% ZnO nanofluids with the theoretical bounds of Nan et al. model. The interfacial thermal resistance used for the upper bound is 0 m^2^K/W and that for the lower bound is 10^-8 ^m^2^K/W [52]. It can be seen clearly that all the thermal conductivity data lie above the bounds predicted by the model of Nan et al.

The comparisons of experimental results with theoretical models convincingly demonstrate that the large enhancements in the thermal conductivities of EG-based ZnO nanofluids are beyond the lower and upper bounds calculated using the models of Maxwell and Nan et al. with and without the interfacial thermal resistance the predictions of the effective medium theory for well-dispersed nanoparticles.

## Conclusions

Ethylene glycol (EG)-based ZnO nanofluids containing no surfactant have been manufactured by one-step physical method using the PWE process. Round-robin tests on thermal conductivity measurements of three samples of EG-based ZnO nanofluids have been conducted and the results have been compared with several theoretical bounds on the effective thermal conductivity of heterogeneous systems. The enhancements of the thermal conductivity of the ZnO nanofluids are beyond the upper and lower bounds of both the Maxwell model and Nan et al. model. Especially, the enhancement of the 5.5 vol.% ZnO nanofluids at 23 C is nearly 25%, while the enhancement predicted by the upper bound of the Maxwell model is at precisely 16.5%. Thus, the discrepancies in the thermal conductivity of the ZnO nanofluids tested in this study cannot be fully explained by the effective medium theory for well-dispersed nanoparticles. Further research is needed to understand and resolve the controversies about contradictory data and new mechanisms of enhanced thermal conductivity in nanofluids.

## Abbreviations

EG: ethylene glycol; H-S: Hashin and Shtrikman; INPBE: International Nanofluid Property Benchmark Exercise; PWE: pulsed wire evaporation; SMEs: small- and medium-sized enterprises; TEM: transmission electron microscopy; THWM: transient hot wire method; ZnO: zinc oxide.

## Competing interests

The authors declare that they have no competing interests.

## Authors' contributions

WHL, CKR, LK, JL, SPJ and SC conceived of the study and participated in its design and coordination. KWL, HYB, GJL, CKK, SWH, YK, DK, SHK, KSH, HJK, HJH, and SHL carried out the experiments. SPJ performed the statistical analysis. SPJ and GJL drafted the manuscript. CJC and JHL checked the equations, figures, and references. SC guided the program and revised the manuscript. All authors read and approved the final manuscript.
